# Supplementary material for: Paracrine Factor Local Gradient‐Generating System for Engineering Perfusable Vascularized Hepatocyte Tissues with Perfusion‐Induced Proliferation
Source: Adv Healthc Mater. 2026 Mar 16;15(19):e04378. doi: 10.1002/adhm.202504378 (PMC13206480; doi:10.1002/adhm.202504378)
Supplement: Supplementary file 1 — Supporting File 1: adhm71032‐sup‐0001‐SuppMat.docx. [file ADHM-15-0-s002.docx]

# Supporting Information

Paracrine Factor Local Gradient-Generating System for Engineering Perfusable Vascularized Hepatocyte Tissues with Perfusion-Induced Proliferation

*Yen-Hsiang Huang, Tadahiro Yamashita, Ryo Sudo**

Supporting Information is available from the Wiley Online Library or from the author.

Figure S1. The process of pre-angiogenesis and subsequent vascularization of hepatocyte tissue. Corresponding phase-contrast images showing the progression of pre-angiogenesis and subsequent vascularization of hepatocyte tissue. During the pre-angiogenesis phase, HUVECs formed angiogenic sprouts under the PFLG generated by hLF cryogels. In the vascularization phase, microvessels penetrated into the hepatocyte tissue. Scale bars, 200 µm.

Figure S2. Luminal structure of microvessels in vascularized hepatocyte tissue. Three-dimensional reconstruction images of vascularized hepatocyte tissue, demonstrating luminal microvessels penetrating into the hepatocyte tissue and recapitulating the architecture of hepatic sinusoids. Blue: cell nuclei (Hoechst 33342), green: F-actin marking hepatocyte tissue (Alexa Fluor 488-phalloidin), red: microvessels (CD31). Scale bars, 50 µm.

Figure S3 Quantitative analysis of urea synthesis from hepatocyte tissues. Urea levels were measured under vascularized (hLF (+), Pre-angio (+)) and non-vascularized (hLF (+), Pre-angio (−)) conditions during a 7-day culture period. Data are presented as mean ± SD (n = 6). **P* < 0.05, ***P* < 0.01, ****P* < 0.001 (one-way ANOVA followed by Tukey’s HSD test).

Figure S4. Growth of vascularized hepatocyte tissue. Phase-contrast images showing progressive growth of vascularized hepatocyte tissue from day 1 to day 5. Scale bars, 200 µm.

Figure S5. Hepatocyte proliferation in non-vascularized hepatocyte tissue. A: Experimental design and timeline showing three different culture conditions: hLF (–) Angio (–), hLF (+) Angio (–), and hLF (+) Angio (+) Penetartraed (–). B: Immunofluorescence images of cell nuclei (blue: Hoechst 33342), F-actin (green: Alexa Fluor 488-phalloidin), HUVECs (red: CD31) and proliferating cell nuclei (yellow: Ki67). White arrowheads indicate Ki67-positive hepatocytes. Red arrowheads indicate non-penetrating microvessels at the boundary of hepatocyte tissue. Scale bars, 50 µm.

Figure S6. Morphological response of rHeps depending on culture media. Phase-contrast images of rHeps in 2D culture comparing EGM-2 + Hep DMEM and EGM-2 + rHep CM over 5 days. Initial seeding density: 5 × 10⁴ cells well^-1^. Scale bars, 200 µm.

Figure S7. Schematic illustrations of microfluidic device design.

Video S1. Luminal structure of microvessels in vascularized hepatocyte tissue. Movie corresponding to Supplementary Fig. 3.

Video S2. Real-time CMTPX excretion by polarized hepatocytes. Movie showing cytoplasmic CMTPX being excreted into bile canaliculi (red: CMTPX

Videos S3. and S4. Fluorescently labeled microbeads perfusion. Time-lapse movies showing the perfusion of fluorescently labeled microbeads (diameter: 1 µm). Sequential fluorescent images were captured using a fluorescence microscope equipped with a high-speed camera.

Video S5. Perfusion of FITC-dextran solution through vascularized hepatocyte tissue. Time-lapse movie was captured using a confocal microscope showing the perfusion of 70 kDa FITC-dextran solution through vascularized hepatocyte tissue. Green: FITC-dextran and F-actin (Alexa Fluor 488-phallidin), red: microvessels (CD31).
